# Exploring the mystical relationship between the Moon, Sun, and birth rate

**DOI:** 10.1186/s12884-024-06654-1

**Published:** 2024-07-01

**Authors:** Ambrogio P. Londero, Serena Bertozzi, Gabriele Messina, Anjeza Xholli, Virginia Michelerio, Laura Mariuzzi, Federico Prefumo, Angelo Cagnacci

**Affiliations:** 1 Academic Unit of Obstetrics and Gynaecology, Department of Neuroscience, Rehabilitation, Ophthalmology, Genetics, Maternal and Infant Health, University of Genoa, Genova, GE 16132 Italy; 2 Obstetrics and Gynecology Unit, IRCCS Istituto Giannina Gaslini, Via Gerolamo Gaslini, 5, Genova, GE 16147 Italy; 3 Breast Unit, Academic Hospital of Udine, Udine, UD 33100 Italy; 4 Beyond Gravity, Zürich, 8052 Switzerland; 5 Academic Unit of Obstetrics and Gynecology, IRCCS Ospedale San Martino, Genoa, 16132 Italy; 6 Institute of Pathologic Anatomy, DAME, Academic Hospital of Udine, Udine, UD 33100 Italy; 7 Department of Neuroscience, Rehabilitation, Ophthalmology, Genetics, Maternal and Infant Health, University of Genoa, Largo Rosanna Benzi, 10, Genova, GE 16132 Italy

**Keywords:** Birth rate, Moon, Sun, Season, Labor, Vaginal delivery

## Abstract

**Objective:**

The Moon has a noticeable influence on the Earth due to its gravity, the most visible manifestation of which are tides. We aimed to see if the Moon’s daily cycle, like the Sun’s, affects the prevalence and incidence of childbirth.

**Methods:**

In this retrospective cohort study, we examined all deliveries at the Academic Hospital of Udine between 2001 and 2019. All consecutive singleton pregnancies with spontaneous labor and vaginal delivery were included.

**Results:**

During the period, 13,349 singleton pregnancies with spontaneous labor and vaginal delivery were delivered in 6939 days. A significantly higher prevalence of deliveries was found with the Moon above the horizon (50.63% vs. 49.37%, *p* < 0.05). Moreover, during the day, there was a significantly higher prevalence of deliveries than during nighttime (53.74% vs. 45.79%, *p* < 0.05). Combining the Moon and Sun altitude, the majority of deliveries were registered when both were above the horizon (27.39% vs. 26.13%, 23.25%, or 23.24%, *p* < 0.05). These findings were confirmed in multivariate analysis after adjusting for parity, gestational age, or season. We found no correlation between birth and the Moon phase.

**Conclusions:**

Our data support the interaction of the Moon and the Sun in determining the time of birth. More research is needed to understand these phenomena and improve our understanding of labor initiation mechanisms.

**Supplementary Information:**

The online version contains supplementary material available at 10.1186/s12884-024-06654-1.

## Highlights- What this paper adds

A significantly higher prevalence of deliveries was found with the Moon above the horizon;

Combining the Moon and Sun, the majority of deliveries were registered when both were above the horizon;

During the summer, we found an increase in the delivery rate.

**Supplementary Information:**

The online version contains supplementary material available at 10.1186/s12884-024-06654-1.

## Introduction

The association between moonlight and birth rate has fascinated people for centuries, with many believing that this celestial phenomenon has a mystical influence on reproduction. Indeed, while a clear association between photoperiod (Sun illumination) and reproduction, including birth rate, has been demonstrated [1–4], the effect of moonlight on birth rate has never been clearly defined [5–12]. In its 24-hour cycle, the Moon influences Earth’s phenomena through its gravitational forces. Along with the Sun’s heating, the Moon’s gravitational forces determine the 24-hour rhythms of ocean tides and the barometric pressure of the atmosphere. Some evidence indicates that a drop in barometric pressure of the atmosphere is associated with the rupture of the amniotic membrane and labor onset, hence influencing birth incidence [13–16].

This study explores the potential impact of solar radiation and 24-hour lunar cycles on human births. Based on the above evidence, our hypothesis suggests that the gravitational forces and solar radiation from the Moon and the Sun, which influence ocean tides and atmospheric pressure, may affect human birth rates. Additionally, the Sun and Moon interaction could regulate hormonal rhythms, such as melatonin secretion, which is known to affect reproductive physiology [2–4, 14].

This study aimed to investigate whether solar radiation and 24-hour lunar cycles can influence the prevalence and incidence rate of human births.

## Methods

### Design, setting, and sample

**Fig. 1:**
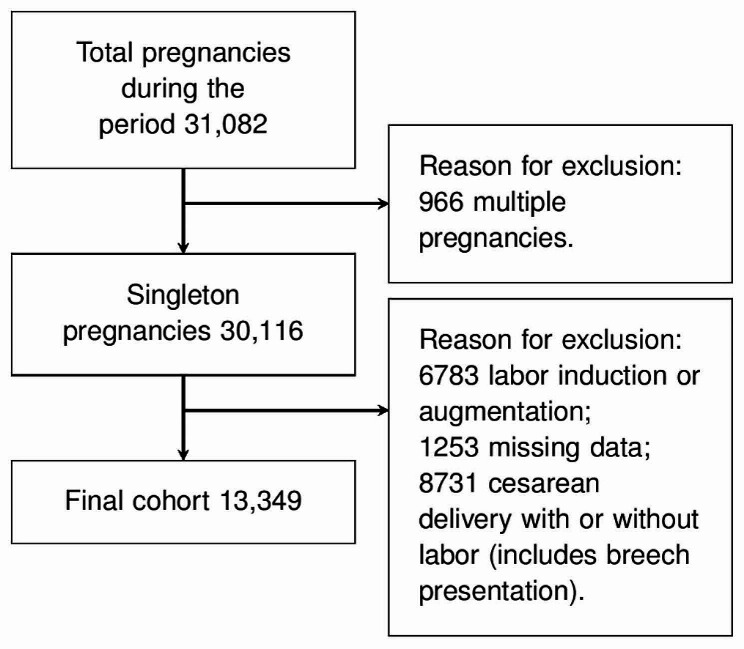
Population selection flowchart

This retrospective study examined all deliveries at the Academic Hospital of Udine between 2001 and 2019. In the analysis, we included all consecutive singleton pregnancies with spontaneous labor from 23 weeks of gestation and vaginal delivery (Fig. 1). We used the induction of labor, the augmentation of labor, cesarean delivery, non-cephalic fetus presentation, and the absence of data as exclusion criteria. The Internal Review Board (IRB) approved this retrospective analysis of anonymized data (25/2018), which followed the policies of the Helsinki Declaration.

### Data collection and measurement

The following data were anonymously extracted from routinary collected data: date and time of delivery, gestational age, parity, neonatal sex, the number of fetuses, onset of labor, and mode of delivery. The onset of labor refers to how labor started, distinguishing between spontaneous labor, labor induced by medical intervention, and pre-labor cesarean delivery. The mode of delivery refers to how the birth was completed, which can be spontaneous vaginal, instrumental vaginal, or cesarean delivery. The date and time of delivery were recorded in local time and transformed into universal time coordinated (UTC) to align with the ephemerides. We calculated according to local latitude and longitude Sun and Moon altitude in reference to the horizon using the suncalc library in R [17]. Using the same library, we extracted daylight (sunrise, day, sunset, night) and Moon phase (waxing crescent, waxing gibbous, waning gibbous, waning crescent) [17]. In our research, sunrise is defined as the initial emergence of the Sun’s upper edge above the horizon, followed by the complete visibility of its lower edge. Sunset commences when the Sun’s lower edge makes contact with the horizon and concludes when it vanishes. The day’s duration spans from the moment the Sun becomes fully visible at sunrise until its initial descent at sunset, while the nighttime period extends from the conclusion of sunset until sunrise. The different phases of the Moon were defined as follows: waxing crescent from the new Moon to the first quarter, waxing gibbous from the first quarter to the full Moon, waning gibbous from the full Moon to the last quarter, and waning crescent from the last quarter to the new Moon. Within our study, we establish the term above the horizon as a binary variable that takes the value of 1 when the Sun or Moon’s altitude exceeds 0 degrees in relation to the horizon. Seasons were attributed according to the northern hemisphere.

### Data analysis

**Fig. 2:**
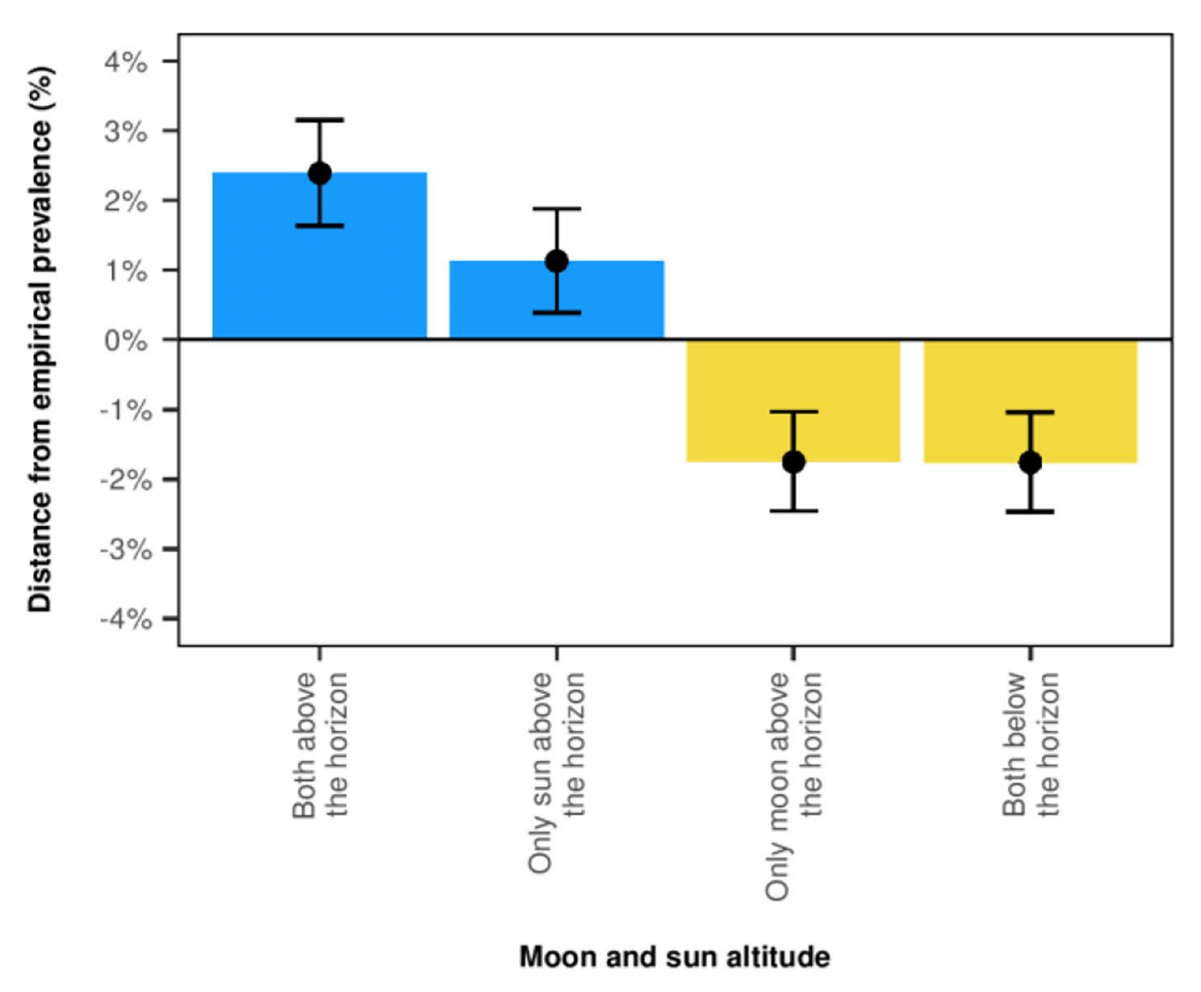
Prevalence of births according to Moon and Sun above the horizon: distance from the expected prevalence and 95% confidence intervals (*p* < 0.05 - this *p*-value refers to the chi-square test of the difference between the births prevalence rates within the plotted groups). The expected prevalence was empirically estimated to be 25% in each category, and the plot shows the difference between the expected and actual prevalence. The cyan bars show a positive difference with a higher prevalence than the expected empirical distribution. Meanwhile, the yellow bars show a negative difference where the prevalence is lower than the expected empirical distribution

R (version 4.2.2) was used to analyze the data, with a two-tailed *p*-value of 0.05 considered significant [18]. All the eligible cases in the considered period were investigated. The statistical analysis was carried out by applying the Kolmogorov-Smirnoff test to determine whether the distribution of continuous variables was parametric. Bivariate analysis for continuous variables was performed using the Wilcoxon test (non-parametric variables) or the t-test (parametric variables). The differences were tested using the Chi-square test or Fisher’s exact test as appropriate for categorical variables. Bivariate and multivariate Poisson regressions were also run to calculate the incidence rate ratio (IRR) and relative 95% confidence intervals (CI.95). Variables were selected according to previous literature and accommodated in multivariate Poisson regressions analysis when the significance level was below 0.05 (parity, gestational age, and season were accommodated in the final multivariate models alongside with Moon and Sun positions, Moon phases and Moon distance). A sensitivity analysis was also performed by randomly imputing the missing values. To prepare Fig. 2, we calculated the empirical distribution of the four categories of the Moon and Sun position (both above the horizon, only the Sun above, only the Moon above, and both below the horizon) during the study period, which was divided into 15-minute intervals. We calculated the Moon and Sun’s relative positions to the horizon for each interval, assessing these positions at 666,145 time points. This analysis revealed an equal 25% distribution across each category, implying an evenly distributed expected birth distribution. Figure 2 depicts the difference, with 95% confidence intervals, between the actual prevalence and expected birth rate distributions previously estimated to be 25% across each category. At the same time, points were calculated for the distance in kilometers between the Earth and the Moon, and the median value of this expected distribution was used to categorize the actual Moon-Earth distance into two categories (first-second quartile of expected distribution vs. third-fourth quartile). Missing data were excluded from this study due to the inclusion criteria but also a sensitivity analysis with random imputation was performed.

## Results

During the considered period, 31,082 pregnancies were delivered in 6939 days. Figure 1 shows the population flowchart and the final analyzed cohort of 13,349 singleton pregnancies with spontaneous labor and vaginal delivery. Table 1 shows pregnancy characteristics. The median maternal age was 32 years, the median gestational age at delivery was 39 weeks, and 48% of women were nulliparous (Table 1).

There was a slight but significantly higher prevalence of deliveries when the Moon was above than below the horizon (50.63% vs. 49.37%, *p* < 0.05), and similar data were observed when the Sun was above the horizon (53.74% at daytime vs. 45.79% at nighttime, *p* < 0.05). The highest prevalence of deliveries (*p* < 0.05) was observed when both the Sun and the Moon were above the horizon (27.3%) vs. only the Sun above the horizon (26.13%), only the Moon above the horizon (23.25%), or both below the horizon (23.24%). The difference between the expected and the actual delivery rate (*p* < 0.05) is reported in Fig. 2 to illustrate the aforementioned differences better. The expected prevalence for each category was empirically estimated to be 25%, and the plot depicts the difference between the expected and actual prevalence. When both the Sun and the Moon were above the horizon, we discovered a significant positive difference with a prevalence greater than the expected empirical distribution. Meanwhile, only the Moon above the horizon or both below the horizon exhibit a negative difference, indicating that the prevalence is lower than the expected empirical distribution. In a sensitivity analysis, we randomly imputed the missing values, and we consistently found the aforementioned significant differences (Supplemental Fig. 1). Moreover, a subgroup analysis showed similar significant differences in the group with spontaneous vaginal birth and in the group with operative vaginal birth (Supplemental Fig. 2A and 2B). These variations were not influenced by parity. We found no evidence that moonlight has any role in the birth prevalence rate (Table 1), while there was an effect of the season with the lowest prevalence of deliveries in autumn and the highest in summer (*p* < 0.05).

The same differences were found when assessing the incidence. The highest number of deliveries happened when the Moon and the Sun were above the horizon. In univariate and multivariate Poisson regression, the IRR showed a significantly lower incidence in all other categories based on the Moon and Sun position relative to the horizon (Table 2). These differences were independent of parity, season, and gestational age. A sensitivity analysis was also performed after randomly imputing the missing values, and we consistently found the forenamed significant differences (Supplemental Table 1).

**Table 1:** Pregnancy characteristics and environmental factors. The table shows the population characteristics. The table presents either the median and interquartile range (IQR) or percentage and absolute values

Variables	Values
**Maternal characteristics**	
Mother’s age (years)	32 (28–35)
Nulliparity	48% (6408/13,349)
**Newborn characteristics**	
Fetal male sex	50.16% (6696/13,349)
Gestational age (weeks)	39 (38–40)
Fetal weight (grams)	3342 (3058–3625)
**Environmental factors**	
Season (*)	
Winter	25.20% (3364/13,349)
Spring	25.04% (3343/13,349)
Summer	25.75% (3437/13,349)
Autumn	24.01% (3205/13,349)
Moon phase (†)	
Waxing Crescent	24.74% (3303/13,349)
Waxing Gibbous	25.09% (3349/13,349)
Waning Gibbous	24.76% (3305/13,349)
Waning Crescent	25.41% (3392/13,349)
Moon distance (Km) (‡)	
First-second quartile of expected distribution	50.02% (6677/13,349)
Third-fourth quartile of expected distribution	49.98% (6672/13,349)
Moon altitude above the horizon (§)	
Moon below the horizon	49.37% (6590/13,349)
Moon above the horizon	50.63% (6759/13,349)
Sunlight (¶)	
Sunrise	0.24% (32/13,349)
Day	53.74% (7174/13,349)
Sunset	0.22% (30/13,349)
Night	45.79% (6113/13,349)
Sun and Moon altitude above the horizon (#)	
Both above the horizon	27.39% (3656/13,349)
Only sun above the horizon	26.13% (3488/13,349)
Only moon above the horizon	23.25% (3103/13,349)
Both below the horizon	23.24% (3102/13,349)

(*) Summer vs. autumn *p* < 0.05; (†) not significant; (‡) not significant; (§) *p* < 0.05; (¶) Day vs. others *p* < 0.05; (#) “both above the horizon” was significantly different from each other category (*p* < 0.05)

**Table 2:** Poisson regression analysis of the daily incidence rates. The table shows the incidence rate ratio (IRR) and the relative 95% confidence interval (CI.95). (*) multivariate model

	IRR (CI.95)	*p*	IRR (CI.95)(*)	*p*(*)
Sun and Moon altitude above the horizon				
Both above the horizon	Reference	---	Reference	---
Only Sun above the horizon	0.954 (0.911–0.999)	< 0.05	0.954 (0.911–0.999)	< 0.05
Only Moon above the horizon	0.849 (0.809–0.890)	< 0.05	0.849 (0.809–0.890)	< 0.05
Both below the horizon	0.848 (0.809–0.890)	< 0.05	0.848 (0.809–0.890)	< 0.05
Gestational age				
Pre-term (< 37 weeks)	Reference	---	Reference	---
Term (≥ 37 weeks)	15.122 (14.095–16.224)	< 0.05	15.122 (14.095–16.224)	< 0.05
Nulliparity	0.923 (0.892–0.955)	< 0.05	0.923 (0.892–0.955)	< 0.05
Season				
Summer	Reference	---	Reference	---
Autumn	0.932 (0.889–0.978)	< 0.05	0.932 (0.889–0.978)	< 0.05
Winter	0.979 (0.933–1.026)	0.376	0.979 (0.933–1.026)	0.376
Spring	0.973 (0.927–1.02)	0.254	0.973 (0.927–1.02)	0.254
Moon phase				
Waxing Crescent	Reference	---	Reference	---
Waxing Gibbous	1.014 (0.966–1.064)	0.573	1.014 (0.966–1.064)	0.573
Waning Gibbous	0.997 (0.950–1.046)	0.893	1.001 (0.953–1.050)	0.980
Waning Crescent	1.031 (0.983–1.082)	0.212	1.027 (0.979–1.077)	0.277
Moon distance (Km)				
First-second quartile of expected distribution	Reference	---	Reference	---
Third-fourth quartile of expected distribution	0.999 (0.966–1.034)	0.965	0.999 (0.966–1.034)	0.965

## Discussion

### Principal findings

Our data show that the birth rate increased significantly when the Sun or Moon was above the horizon. The 24-hour delivery incidence was highest when both the Moon and the Sun were above the horizon. Moreover, we found no relationship between birth rate and the Moon’s illumination or distance.

## Results in the context of what is known

The influence of the lunar cycle on births has been widely studied with contrasting results [5–12]. The correlation sought between birth incidence and synodic month (lunar phase, which corresponds to moon illumination percentage) or sidereal month (lunar distance) found by some [9, 11, 12] was not confirmed by others [12]. We also found no correlation between the synodic or sidereal month and birth. None of those previous studies assessed the Moon’s shorter cycle, determined by the daily-repeated Moon’s rise and set due to Earth’s rotation on its axis, independent of people’s capability to see the Moon when masked by sunshine. While it is unclear how moonlight can act, it is clear that the Moon above the horizon creates a gravity attraction capable of influencing sea movements. Ocean tides are caused primarily by the Moon’s gravitational pull [19] with an oscillation related to the lunar day (time between successive lunar transits) of about 24 h 51 min. The Moon’s gravitational pull contributes to the Sun’s heating to create a 24-hour cycle of atmospheric tides with the highest barometric pressure during the day and with the Moon and Sun above the horizon and lowest barometric pressure at night and the Moon below the horizon [20–23].

Previous research found that barometric pressure drop is associated with labor onset and amniotic membrane rupture [14–16]. This finding was not confirmed in another study [10]. Yet none of these studies evaluated the daily rhythm of barometric pressure due to atmospheric tides. Our analysis shows that most births occur during the day. An influence of photoperiod and melatonin on labor initiation at night and birth at day has been suggested [2–4], but as our data indicate, modifications of this environmental phenomenon due to the Moon cycle may also be implicated [14].

Seasonal modification of the birth rate herein reported reflects previously described seasonality of human reproduction [3, 24–27].

### Clinical implications

The evidence of the effect of the Moon on birth indicates an impact on human biological functions that has never been reported before and may have implications in other medical fields.

### Research implications

The possible effect of the Moon on birth should be further explored in other conditions, possibly affecting the barometric pressure of the atmosphere. In addition, the biological mechanisms influenced by the Moon need to be understood to have a better knowledge about birth.

### Strengths and limitations

The main limitation of the present study is its retrospective nature. In fact, the data only presented information on the delivery timing and not on the onset of labor, its duration, the timing of the rupture of the amniotic membranes, and the uterine contractile activity. Although all of this information could be useful in better understanding the mechanisms that connect humans to the environment in which they live, it is important to note that while analyzing the onset of labor is highly desirable, it presents extraordinary challenges. Additionally, the study’s limitations include the need for more detailed information on atmospheric conditions throughout the day, particularly variations in barometric pressure. This information is also useful for validating the discussed working hypotheses. Another limitation is the human intervention before and during labor, which can impair the correlations between labor and the associated factors, particularly given the ongoing rise in cesarean deliveries and labor inductions [28, 29]. Although some strategies to reduce medical interventions in labor have recently been implemented, the number of medical interventions is still high [30]. In our study, we included only vaginal deliveries from spontaneous labor to minimize the effect of medical interventions. According to previous literature, other factors, such as season and parity, could influence the birth rate. We performed a multivariate analysis with the available data to avoid these possible confounding. Another potential study limitation is the amount of missing data. However, there was a low rate of missing information, and analyses with missing data imputation yielded the same results. Meanwhile, the main strength of the present study is the analysis of the daily change in the Sun and Moon altitude as factors correlated to the birth rate. In fact, previous studies did not assess the influence of the Moon’s daily cycle but focused only on synodic and sidereal months.

## Conclusions

Our data support the presence of a small but significant effect of the Sun and Moon’s interaction with birth rate. Further studies are required to understand these phenomena and improve our knowledge of labor initiation mechanisms.

### Electronic supplementary material

Below is the link to the electronic supplementary material.

Supplementary Material 1

Supplementary Material 2

Supplementary Material 3

## Data Availability

The data that support the findings of this study are available, but restrictions apply to the availability of these data, which were used under license for the current study, and so are not publicly available. Data are, however, available from the corresponding author upon reasonable request and with permission of the Internal Review Board (ambrogiopiertro.londero@unige.it or ambrogiopietro.londero@asufc.sanita.fvg.it).

## Declarations

### Condensation

Our data show that the birth rate increased significantly when the Sun or Moon was above the horizon. When both the Moon and the Sun were above the horizon, the 24-hour delivery incidence was highest. Furthermore, we discovered no correlation between delivery incidence and the Moon’s phases.

## Abbreviations

CI.95: confidence intervals
IRR: incidence rate ratio
UTC: universal time coordinated

## References

[1] Mark PJ, Crew RC, Wharfe MD, Waddell BJ (2017). Rhythmic three-part harmony: the Complex Interaction of maternal, placental and fetal Circadian systems. J Biol Rhythms.

[2] Moore TR, Iams JD, Creasy RK, Burau KD, Davidson AL (1994). Diurnal and gestational patterns of uterine activity in normal human pregnancy. The uterine activity in pregnancy Working Group. Obstet Gynecol.

[3] Cagnacci A, Soldani R, Melis GB, Volpe A (1998). Diurnal rhythms of labor and delivery in women: modulation by parity and seasons. Am J Obstet Gynecol.

[4] Cagnacci A, Soldani R, Yen SS (1997). Contemporaneous melatonin administration modifies the circadian response to nocturnal bright light stimuli. Am J Physiol.

[5] Ghiandoni G, Seclì R, Rocchi MB, Ugolini G (1997). [Incidence of lunar position in the distribution of deliveries. A statistical analysis]. Minerva Ginecol.

[6] Ghiandoni G, Seclì R, Rocchi MB, Ugolini G (1998). Does lunar position influence the time of delivery? A statistical analysis. Eur J Obstet Gynecol Reprod Biol.

[7] Arliss JM, Kaplan EN, Galvin SL (2005). The effect of the lunar cycle on frequency of births and birth complications. Am J Obstet Gynecol.

[8] Romero Martínez J, Guerrero Guijo I, Artura Serrano A (2004). [The moon and delivery]. Rev Enferm.

[9] Staboulidou I, Soergel P, Vaske B, Hillemanns P (2008). The influence of lunar cycle on frequency of birth, birth complications, neonatal outcome and the gender: a retrospective analysis. Acta Obstet Gynecol Scand.

[10] Morton-Pradhan S, Bay RC, Coonrod DV (2005). Birth rate and its correlation with the lunar cycle and specific atmospheric conditions. Am J Obstet Gynecol.

[11] Gudziunaite S, Moshammer H (2022). Temporal patterns of weekly births and conceptions predicted by meteorology, seasonal variation, and lunar phases. Wien Klin Wochenschr.

[12] Morales-Luengo F, Salamanca-Zarzuela B, Marín Urueña S, Escribano García C (2020). Caserío Carbonero S. [External influences on birth deliveries: Lunar gravitational and meteorological effects]. Pediatr (Engl Ed).

[13] Wheeler ML, Oyen ML (2020). Premature rupture of membranes and severe Weather systems. Front Physiol.

[14] King EA, Fleschler RG, Cohen SM (1997). Association between significant decrease in barometric pressure and onset of labor. J Nurse Midwifery.

[15] Akutagawa O, Nishi H, Isaka K (2007). Spontaneous delivery is related to barometric pressure. Arch Gynecol Obstet.

[16] Yackerson N, Piura B, Sheiner E (2008). The influence of meteorological factors on the emergence of preterm delivery and preterm premature rupture of membrane. J Perinatol.

[17] Thieurmel B, Elmarhraoui A, Suncalc. Compute Sun Position, Sunlight Phases, Moon Position and Lunar Phase. 2022.

[18] R Core Team (2022). R: a Language and Environment for Statistical Computing.

[19] Volland H (1988). Atmospheric Tidal and Planetary waves.

[20] Haurwitz B (1964). Atmospheric tides: these oscillations are caused by the gravitational pull of sun and moon and by the sun’s thermal effects. Science.

[21] Huang NE, Shen Z, Long SR, Wu MC, Shih HH, Zheng Q (1998). The empirical mode decomposition and the Hilbert spectrum for nonlinear and non-stationary time series analysis. Proc R Soc Lond A.

[22] Zhong D, Quan H. Analysis of Earth Gravity Tide Signal Based on EMD and Information Extraction:. In 4th International Conference on Machinery, Materials and Computing Technology. Atlantis Press, Hangzhou, China. 2016; 836–840.

[23] Lieberman RS, Harding BJ, Heelis RA, Pedatella NM, Forbes JM, Oberheide J. Atmospheric Lunar Tide in the low latitude Thermosphere-Ionosphere. Geophys Res Lett. 2022;49. 10.1029/2022GL098078 PMC9286538 35865010

[24] Hoffmann F, Kawiani D (1976). [Seasonal variations in the birth rate and conception rate within the last 200 years (author’s transl)]. Geburtshilfe Frauenheilkd.

[25] Nenko I, Briga M, Micek A, Jasienska G (2022). From January to June: birth seasonality across two centuries in a rural Polish community. Sci Rep.

[26] Cesario SK (2002). The Christmas Effect and other biometeorologic influences on childbearing and the health of women. J Obstet Gynecol Neonatal Nurs.

[27] Walfisch A, Kabakov E, Friger M, Sheiner E (2017). Trends, seasonality and effect of ambient temperature on preterm delivery. J Matern Fetal Neonatal Med.

[28] Fruscalzo A, Rossetti E, Londero AP. Trial of Labor after three or more previous cesarean sections: systematic review and Meta-analysis of Observational studies. Z Geburtshilfe Neonatol; 2022. 10.1055/a-1965-4125 36455615

[29] Haavaldsen C, Morken NH, Saugstad OD, Eskild A. Is the increasing prevalence of labor induction accompanied by changes in pregnancy outcomes? An observational study of all singleton births at gestational weeks 37–42 in Norway during 1999–2019. Acta Obstet Gynecol Scand. 2022. 10.1111/aogs.14489 PMC9889324 36495002

[30] Zipori Y, Grunwald O, Ginsberg Y, Beloosesky R, Weiner Z (2019). The impact of extending the second stage of labor to prevent primary cesarean delivery on maternal and neonatal outcomes. Am J Obstet Gynecol.

