# Supplementary material for: Exploring the mystical relationship between the Moon, Sun, and birth rate
Source: BMC Pregnancy Childbirth. 2024 Jul 1;24:454. doi: 10.1186/s12884-024-06654-1 (PMC11218357; doi:10.1186/s12884-024-06654-1)
Supplement: Supplementary file 1 — Supplementary Material 1 [file 12884_2024_6654_MOESM1_ESM.pdf]

Supplemenatl Table 1. Poisson regression analysis of the daily incidence rates. The missing values in these models were randomly imputed. The table shows the incidence rate ratio (IRR) and the relative 95% confidence interval (CI.95). (*) multivariate model.

	IRR (CI.95)	p	IRR (CI.95)(*)	p(*)
Sun and Moon altitude above the horizon				
Both above the horizon	Reference	---	Reference	---
Only Sun above the horizon	0.954 (0.912 - 0.998)	<0.05	0.954 (0.912 - 0.998)	<0.05
Only Moon above the horizon	0.836 (0.798 - 0.875)	<0.05	0.836 (0.798 - 0.875)	<0.05
Both below the horizon	0.836 (0.798 - 0.875)	<0.05	0.836 (0.798 - 0.875)	<0.05
Gestational age				
Pre-term (<37 weeks)	Reference	---	Reference	---
Term (≥37 weeks)	15.109 (14.112 - 16.177)	<0.05	15.109 (14.112 - 16.177)	<0.05
Nulliparity	0.914 (0.884 - 0.945)	<0.05	0.914 (0.884 - 0.945)	<0.05
Season				
Summer	Reference	---	Reference	---
Autumn	0.940 (0.897 - 0.985)	<0.05	0.940 (0.897 - 0.985)	<0.05
Winter	0.978 (0.933 - 1.024)	0.334	0.978 (0.933 - 1.024)	0.334
Spring	0.966 (0.923 - 1.012)	0.146	0.966 (0.923 - 1.012)	0.146
Moon phase				
Waxing Crescent	Reference	---	Reference	---
Waxing Gibbous	1.014 (0.968 - 1.063)	0.551	1.014 (0.968 - 1.063)	0.551
Waning Gibbous	1.006 (0.960 - 1.054)	0.811	1.006 (0.960 - 1.054)	0.811
Waning Crescent	1.030 (0.983 - 1.079)	0.208	1.030 (0.983 - 1.079)	0.208
Moon distance (Km)				
First-second quartile of expected distribution	Reference	---	Reference	---
Third-fourth quartile of expected distribution	0.999 (0.966 - 1.034)	0.965	0.999 (0.966 - 1.034)	0.965
