# Supplementary figures and images for: Exploring the mystical relationship between the Moon, Sun, and birth rate
Source: BMC Pregnancy Childbirth. 2024 Jul 1;24:454. doi: 10.1186/s12884-024-06654-1 (PMC11218357; doi:10.1186/s12884-024-06654-1)

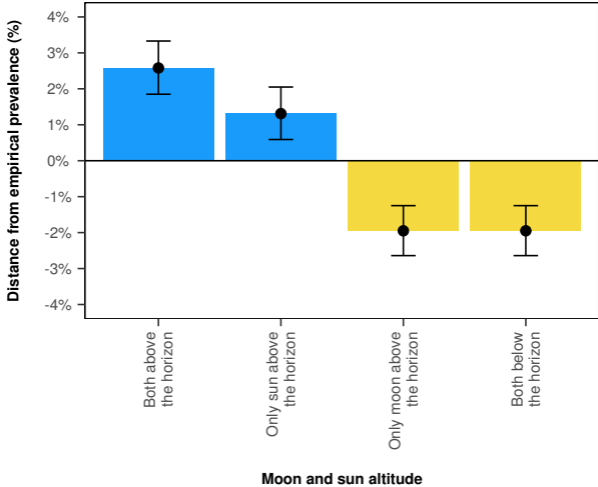

Supplement: Supplementary file 2 — Supplementary Material 2 [file 12884_2024_6654_MOESM2_ESM.pdf]

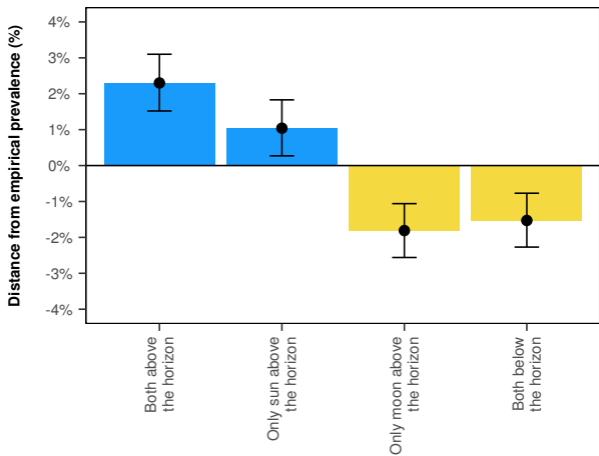

**A**

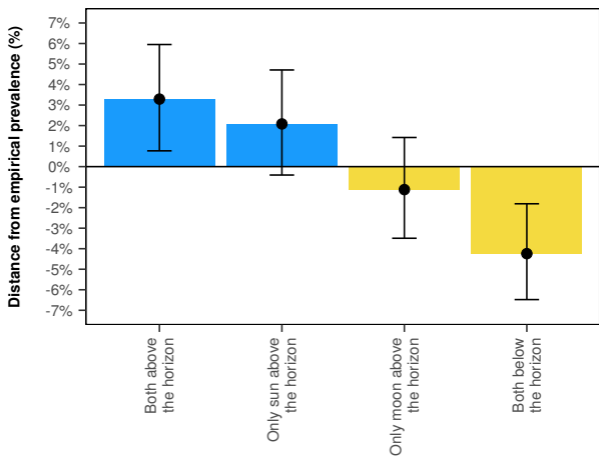

**B**

Supplement: Supplementary file 3 — Supplementary Material 3 [file 12884_2024_6654_MOESM3_ESM.pdf]
